# Editorial: Mucosal vaccines for the induction of antimicrobial immunoglobulin A

**DOI:** 10.3389/fimmu.2024.1270600

**Published:** 2024-02-27

**Authors:** Reiko Shinkura

**Affiliations:** Institute for Quantitative Biosciences, The University of Tokyo, Tokyo, Japan

**Keywords:** mucosal vaccine, immunoglobulin A, mucosal immunity, mucosal adjuvant, pandemic

IgA antibodies that work in the mucosa, such as intestinal IgA antibodies, are known to have a wide range of antigen specificities, allowing a single type of antibody to respond to multiple antigens. Note that unlike monomeric IgA found in serum, the intestinal environment harbors dimeric or tetrameric forms of IgA, known as secreted IgA (SIgA).

Recently, many papers have shown that mucosal IgA antibodies can deal with a wider range of variants than IgG antibodies, even against the pandemic SARS-CoV2 experienced. In addition, it has become clear that intestinal IgA antibodies, while recognizing multiple antigens, bind to pathogens selectively, with different effects depending on the type of partner, although the molecular mechanisms are still unclear. However, the importance of IgA antibodies in the control of gut microbiota is attracting increasing attention, especially as it becomes clear that the gut microbiota is strongly associated with many diseases. Moreover, the presence of only small amounts of IgA antibodies in germ-free mice indicates that the production of secretory IgA antibodies is induced by stimulation of the microbiome, an important interaction between the microbe and the host immune system.

This Research Topic has focused on the question of how to strengthen the immunity of the mucosa, which is the portal of entry for pathogens into the body. In particular, research has focused on mucosal vaccines that efficiently induce mucosal IgA antibodies that can block pathogens before they enter the body at the mucosal surface. IgA antibodies are thought to be responsible for the overall balance of the bacterial flora, as they screen for bacteria and keep bad bacteria in check and good ones out. However, the mechanisms underlying the selection of viruses by IgA antibodies remain largely unexplored.

The research contained in this Research Topic is an important area that should continue to be carried out in the fight against pathogens, which is expected to continue in the future.

Hopefully, future research will progress further and deeper to combat the next pandemic.

## Author contributions

RS: Writing – original draft.

